# The suitability of using death certificates as a data source for cancer mortality assessment in Turkey

**DOI:** 10.3325/cmj.2012.53.480

**Published:** 2012-10

**Authors:** Tumer Ulus, Eray Yurtseven, Sabanur Cavdar, Ethem Erginoz, M. Sarper Erdogan

**Affiliations:** Public Health Department, Cerrahpasa Medical Faculty, Istanbul University, Istanbul, Turkey

## Abstract

**Aim:**

To compare the quality of the 2008 cancer mortality data of the Istanbul Directorate of Cemeteries (IDC) with the 2008 data of International Agency for Research on Cancer (IARC) and Turkish Statistical Institute (TUIK), and discuss the suitability of using this databank for estimations of cancer mortality in the future.

**Methods:**

We used 2008 and 2010 death records of the IDC and compared it to TUIK and IARC data.

**Results:**

According to the WHO statistics, in Turkey in 2008 there were 67 255 estimated cancer deaths. As the population of Turkey was 71 517 100, the cancer mortality rate was 9.4 per 10 000. According to the IDC statistics, the cancer mortality rate in Istanbul in 2008 was 5.97 per 10 000.

**Conclusion:**

IDC estimates were higher than WHO estimates probably because WHO bases its estimates on a sample group and because of the restrictions of IDC data collection method. Death certificates could be a reliable and accurate data source for mortality statistics if the problems of data collection are solved.

Due to the rapid aging of populations, diet, use of tobacco and other substances (cannabis etc), and infectious agents, cancer remains a major cause of morbidity and mortality worldwide. Incidence rates of many cancers could increase substantially in the future, with up to 15 million new cases expected in 2020. Most of them will occur in developing countries (1,2). Although the overall incidence of cancer in developing countries is still lower than in the developed world, the mortality rate of cancer is comparable (3). In order to adequately respond to the growing cancer pandemic in developing countries, we should gather information, use that information to determine priorities, and implement appropriate initiatives (4).

Developing countries largely lack effective cancer surveillance and control, adequate health care, funding, and coverage at the national level. The magnitude of the cancer burden is poorly understood owing to a lack of surveillance and monitoring systems for cancer risk factors and a lack of documentation of cancer incidence and mortality. Surveillance of cancer, as well as a well-functioning data collection mechanism, is critical to the implementation and evaluation of primary and secondary prevention (2) and to understanding of the cancer burden (5). The “gold-standard” in cancer surveillance is a population-based cancer registry. Through this registry, information on new cancer cases could be systematically and continually collected. The data from such a registry would allow for the calculation of cancer incidence rates when used with corresponding census data. Such cancer registries require sustained commitments and trained personnel, which are also lacking in many countries (5).

Crucial steps in cancer surveillance are improving infrastructure and training capacity for gathering of cancer incidence, prevalence, and mortality data. Accurate statistics on cancer occurrence and outcome are essential both for the research purposes and for the planning and evaluation of programs for cancer control (6). However, statistics on cancer incidence and mortality available in developing countries are often incomplete. This creates a challenge to policy and resource allocation, as epidemiologic data are crucial for identification of cancer risk factors and etiologies (7).

Several international agencies have published statistics on cancer mortality rates and incidence, including those from developing countries. The International Agency for Research on Cancer (IARC), a component of the WHO (6), periodically collects data and produces estimates of cancer incidence and mortality for all countries, whether a cancer registry exists or not. These estimates are updated periodically on GLOBOCAN projects (5).

GLOBOCAN is a project conducted by IARC, which presents the estimates of cancer incidence and mortality from major types of cancers of 184 countries or territories of the world (8). Cancer Incidence in Five Continents (CI5), an IARC publication, provides information on the incidence of cancer recorded by cancer registries worldwide. CI5 is the product of the collaboration between the IARC and the network of cancer registries worldwide, represented by the International Association of Cancer Registries. Data from Turkey are provided by the Cancer Control Department of the Ministry of Health (9) and refer to the provinces of Izmir and Antalya. The population-based registry in Turkey was started in Izmir in 1991 and is run by the Cancer Control Department of the Ministry of Health. It has been extended to other provinces that are thought to be representative of Turkey as a whole (8). 

Incidence data are obtained from population-based cancer registries, and mortality data from vital registration systems, where the fact and “underlying” cause of death are certified by a medical practitioner (8). In Turkey, death certificates are issued by municipality physicians and family physicians. Hospital doctors issue the death certificate if the person dies in the hospital. Death certificates represent also the permission for burial. Copies of death certificates are sent both to the Provincial Directorates of Cemeteries under Municipalities and to the Community Health Centers of the Provincial Directorate of Health, which then forward the copy to the Turkish Statistical Institute (TUIK). TUIK is the institution that collects and publishes official economic, social, and demographic data (10). It also collects death statistics. While incidence data are generally published with some delay as they require time to be compiled, mortality data are easier to generate, especially if they are kept in a computerized system.

In this study, we compared the quality of the 2008 cancer mortality data of the Istanbul Directorate of Cemeteries with the 2008 data of IARC and TUIK, and discussed the suitability of using this databank for estimations of cancer mortality in the future.

## Material and methods

We used 2008 and 2010 death records of the Istanbul Directorate of Cemeteries. A request was made to the Directorate and in April of 2011 the data were provided. In the Directorate, data from death certificates handwritten by physicians are transferred to computers. The personnel responsible for entering the data are non-medical staff. The variables that are taken into consideration are name, sex, age, address, time of death, cause of death, and burial place. In Turkey, it is not legal to perform burial without a death certificate or anywhere other than in cemeteries. We may therefore assume that the Directorate data are comprehensive. As the same data are also kept in the Municipality, we requested that it should be cross checked and any irregularities accounted for. The TUIK mortality data can be accessed on line (10).

We accessed the 2008 IARC GLOBOCAN data in May 2011. The number of cancer deaths in Turkey in 2008 was estimated from the incidence estimates calculated from the data provided by the cancer registry offices from two 2 cities, which operate compatible with the standards of IARC, and site-specific survival using the gross domestic product (GDP) after having made some corrections. GDP method estimates the relationship between cancer-specific 5-year relative survival and country-specific GDP per capita based on the assumption that cancer survival is reasonably correlated with the level of GDP (11). The overall number of deaths was corrected for under-reporting or incompleteness using percentages provided by the WHO. When the category ill-defined cause of death exceeded 3% of the total causes of death, the excess was partitioned by sex and age into cancer deaths and other specific causes of death. The corrected cancer deaths category was partitioned into cancer-specific categories using proportions from the non-corrected data. The number of cancer cases and cancer deaths was scaled to the estimated WHO total number of cancer deaths for 2008. WHO extracted national population estimates for 2008 from the United Nations (UN) population division, the 2008 revision (United Nations, Population Division, World Population Prospects, the 2008 revision, *http://www.un.org/*).

## Results

According to the WHO statistics, the estimated number of cancer deaths in 2008 was 67 255. As in the same year the population of Turkey was 71 517 100, the cancer mortality rate was 9.4 per 10 000. The four most mortal cancers were trachea, bronchus, and lung; stomach; colon and rectum; and breast cancer. These accounted for 47.7% of total cases (Table 1).

**Table 1:** World Health Organization estimates of cancer deaths in Turkey by cause, 2008

Malignant neoplasms	Deaths		Mortality rate (100 000)
	n	%	
Trachea, bronchus, lung	15 101	22.45	21.11
Stomach	7769	11.55	10.86
Colon and rectum	4949	7.30	6.92
Breast cancer	4311	6.40	6.02
Lymphomas, multiple myeloma	3516	5.22	4.91
Leukemia	2931	4.35	4.09
Bladder	2892	4.30	4.04
Esophagus	2878	4.27	4.02
Prostate	2853	4.24	3.98
Pancreas	1972	2.93	2.75
Liver	1536	2.28	2.14
Ovary	1247	1.85	1.74
Mouth and oropharynx	1232	1.83	1.72
Cervix uteri	556	0.84	0.77
Corpus uteri	519	0.77	0.72
Melanoma and other skin	401	0.60	0.56
Other	12 592	18.82	17.6
Total	67 255	100.00	94.04

According to the statistics produced by the Istanbul Directorate of Cemeteries, the number of cancer deaths in Istanbul in 2008 was 7500 (Table 2). As the population of Istanbul in the same year was 12 697 164, the cancer mortality rate was 5.97 per 10 000, by simply dividing the number of deaths with the population of Istanbul. If the cancer mortality rate for Turkey estimated by WHO was weighed by 2008 population of Istanbul relative to Turkey, the number of cancer deaths in Istanbul would be 11 814. In the same year, the number of death cases recorded by TUIK was 8640, which does not comprise the dead people transported to the city from the family’s place of origin. By using the same WHO classification, the four most prevalent types of cancer accounted for 62.93% of the total of cancer deaths.

**Table 2:** The distribution of cancer deaths in Istanbul by cause, 2008*

Malignant neoplasms	Deaths		Mortality rate (100 000)
	n	%	
Trachea, bronchus, lung	2918	38.90	23.21
Stomach	737	9.82	5.86
Colon and rectum	706	9.41	5.61
Breast	435	5.80	3.46
Pancreas	391	5.21	3.11
Liver	284	3.78	2.25
Prostate	279	3.72	2.21
Lymphomas, multiple myeloma	179	2.38	1.42
Bladder	161	2.14	1.28
Ovary	149	1.98	1.18
Leukemia	115	1.53	0.91
Melanoma and other skin	103	1.37	0.81
Corpus uteri	88	1.17	0.7
Esophagus	66	0.88	0.52
Cervix uteri	18	0.24	0.14
Mouth and oropharynx	17	0.22	0.13
Other	854	11.45	6.79
Total	7500	100.00	59.67

*Obtained from the Istanbul Directorate of Cemeteries.

The number of cancer deaths recorded by the Istanbul Directorate of Cemeteries in Istanbul in 2010 was 6896, while WHO and TUIK have not yet published any data for Turkey beyond 2008.

## Discussion

The number of cancer deaths in Istanbul was 7500 according to the Istanbul Directorate of Cemeteries, 11 814 according to the WHO (11), and 8640 according to TUIK. One of the reasons for the discrepancy is the influence of the variability of cancer risk in different populations. However, notwithstanding the differences, the prevalence of types of cancers seems to be entirely accurate. The first four cancers on the WHO list were exactly the same as those listed by the Directorate of Cemeteries. Similarly, in most of the developing countries, most frequent types of cancer were lung, breast, stomach, colorectal, and liver cancers (12-14).

WHO collects and presents mortality statistics worldwide. The data sets from different nations are not of the same quality. In some countries, coverage of the population is incomplete, so mortality rates are low or the quality of cause of death information is poor. While almost all the European and American countries have comprehensive death registration systems, most of the developing countries do not. Official Turkish statistics are presented by TUIK, but IARC receives the cancer data from the Cancer Control Department of the Ministry of Health, which is the specialized center for registration in Turkey.

TUIK obtains mortality data through Provincial Health Directorates from the death certificates filled out by municipality doctors and family physicians, and in some cases, by hospital doctors. The key task in filling out the certificates is to record the cause of death correctly, for the purpose of which there are 4 lines, marked a-d. The top line should state the “end cause,” with the following lines going down to the “main cause.” Diseases such as cancer should be recorded on the “d” line, with the immediate cause, for example sepsis or anaphylaxis, on the “a” line. As a result, even when doctors are clear about the cause of death, they may not record it correctly in terms of order. Data on death events occurring in province and district centers are classified by TUIK in compliance with international standards and in accordance with the International Disease Categories (ICD-8), which list 50 and 150 causes for tabulation of mortality as required by the WHO. Mortality statistics are evaluated and presented annually to the public.

The other institution that collects death certificates and keeps these records is the Directorates of the Cemeteries under Municipalities. Although in both cases, death certificates are the only source used to extract the data, there is a difference between the TUIK statistics and those of the Directorates of Cemeteries. TUIK specializes in processing statistics, so its personnel are more likely to extract the key information even when it is not in the correct order. While staff working on the databanks of the cemeteries may simply transfer information from the “d” line of the certificate, the personnel in TUIK are trained to seek the “main cause” among the given causes. Because of this, the calculation of cancer registry from death certificates is widely accepted as non-reliable. IARC claims in its World Cancer Report 2003 that any rate calculated from death certificates would be below the actual figure, which is the case in our study.

The WHO data cover entire national populations but more often they cover smaller, subnational areas or only major cities, particularly in developing countries. National mortality rates in some countries have a correction factor applied to account for known and quantified underreporting of deaths. Rates for the missing sites are computed using proportions from mortality files provided by the reliable part of cancer registries. When national mortality data are unavailable or are of poor quality, regional mortality rates derived from the data of one or more cancer registries covering a part of the country are used and mortality is estimated from incidence rates, using country/region-specific survival (8). The WHO data may also contain an error, which is assumed to be tolerable. The cancer morbidity figures from Sample Registry System were low compared with the cancer registry of Health Ministry figures (15).

Registries collect the data on cancer deaths from three main sources: municipal health directorates through physicians who report the death; hospital medical records; and active follow-up through telephone, postal enquiries, and house visits. There are many reasons for under-registration of cancer deaths in cancer registries: the death might have occurred outside the area of registration, it may not have been registered at the municipal health directorate, or the cancer may have been overlooked; if the cancer has long survival the information about cancer could have been totally forgotten. Also, migration plays an important role, and cause of death may be erroneously reported as the last cause of death instead of the main reason (16).

Nonetheless, death certificates that are normally ignored when death rates are calculated have the potential to provide an important supplementary source of information for cancer registries. As far as incidence statistics are concerned, they function as a means of capturing information on cases that were omitted in the registration process. Cancer registries may evaluate the completeness of their work on the basis of the proportion of incident cancers that first come to the registry’s attention via a Death Certificate Notification of cancer (DCN) (17). In that case the DCN cases are measured against the Death Certificate Only (DCO) cases, for which no other information than a death certificate mentioning cancer could be obtained. The DCO cases fail to show any evidence (disease report, official document, etc.) other than death certificate. An elevated DCO percentage is suggestive of incompleteness. But, of course, this must be interpreted in the light of local circumstances, since the quality of the already existing death certificates may be very poor, as is the case in some developing countries. In the Volume IX of CI5 of IARC, data sets with less than 20% of DCO cases were considered for the evaluation (18).

On the other hand, another measure of validity could be the proportion of cancers for which no other information other than a death certificate mentioning cancer can be obtained (18). The information on death certificates is known to suffer from lack of accuracy, or lack of precision, compared with that obtained from clinical or pathology records (19).

The IARC data may contain some errors because they are based upon a sampling system. It is a generalized estimation based on a sample group, so countries should establish more reliable data collection systems. For mortality statistics, death certificates can be a more reliable and accurate data source, if sufficient attention is paid to them. The quality of mortality data could be enhanced by establishing computerized systems, checking the data input, and training the physicians and system analysts.
